# The Antimicrobial Properties of Technical Lignins and Their Derivatives—A Review

**DOI:** 10.3390/polym16152181

**Published:** 2024-07-31

**Authors:** Diana Carolina Reyes, Zhengxin Ma, Juan Jose Romero

**Affiliations:** 1 Animal and Veterinary Sciences, University of Maine, Orono, ME 04469, USA; dcr232@cornell.edu; 2 Animal Science, Cornell University, Ithaca, NY 14850, USA; 3 Molecular and Biomedical Sciences, University of Maine, Orono, ME 04469, USA; zhengxin.ma@maine.edu

**Keywords:** technical lignins, antimicrobial, applications

## Abstract

Lignin represents one of the most abundant plant-derived polymers. It is mostly present in the cell wall, and its primary role is to provide mechanical support to the plant. Chemical processes during wood-pulping yield diverse technical lignins with distinct characteristics. Due to their complex and variable nature, technical lignins are often undervalued and are mainly used as burning fuel in mills. However, various technical lignins have been shown to possess antimicrobial properties. Consequently, there is an increasing interest in understanding the properties and conditions that underlie their antimicrobial characteristics and how we can utilize them for practical applications. This review, for the first time, comprehensively summarized the antimicrobial activities of technical lignins and their potential antimicrobial applications.

## 1. Introduction

Lignin is the most abundant natural terrestrial polymer after cellulose and chitin. It is one of the main constituents of the plant cell wall, providing mechanical support, controlling water conduction, and protecting the plant from biochemical degradation [1]. Approximately 50 million Mg of commercial purified lignin (i.e., technical lignins) are separated from wood during pulp and papermaking processes each year worldwide [2]. However, due to its complex nature, only a small portion (1–2%) of this material is processed into valuable byproducts, with the rest being incinerated [3]. The source of lignin (hardwood vs. softwood) and the extraction conditions affect lignin’s structure and chemical properties, which in turn influence its end-use applications. The four major processes used for technical lignin production include sulfite, Kraft, soda, and organosolv pulping [4]. Technical lignins are currently used commercially as binders, additives for concrete mixtures, dispersants, and feed and food additives [5].

Among the commercially available technical lignins, lignosulfonates and Kraft lignins have been reported to exhibit antibacterial [6], antifungal [7], and antiviral activities [8], and prebiotic activities [9]. Furthermore, recent research in the development of novel biopolymers with microbicidal properties has led to the development and assessment of lignin-derived biomaterials, including dealkaline lignin-based hydrogels [10], nanocomposite fibers made from alkali Kraft lignin, and nanoparticles synthesized by dissolving alkali Kraft lignin into ethylene glycol [11]. Successful modifications that boost the antimicrobial properties of technical lignins will revamp the value of forest bioproducts, which will benefit greatly the areas heavily dependent on forest resources. Understanding the antimicrobial properties and mechanisms of technical lignins will lead to innovative solutions for combating bacterial infections, enhancing animal health and food safety, and mitigating environmental contaminations. Currently, the modes of action of technical lignin antimicrobial activities are not well understood, as different classes of technical lignins may have different mechanisms. In addition, the wide array of technical lignins and their derivatives, as well as the divergent methodologies used to assess their antimicrobial activity, create a challenge for direct comparisons between studies. This review aims to provide a thorough exploration of the antimicrobial attributes exhibited by technical lignins. It serves to consolidate, for the first time, the existing research on assessing the antimicrobial properties of technical lignins and their derivatives, structured around their chemical properties.

## 2. Native Lignin Chemical Properties

**Figure 1:**
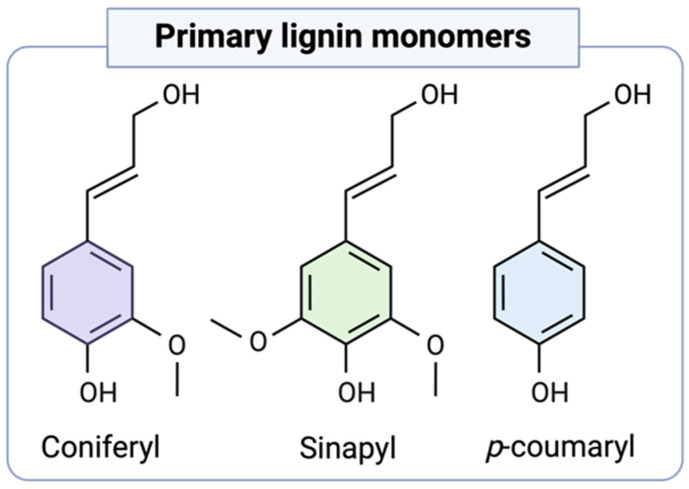
Primary lignin monolignols.

Lignin is defined as a complex phenolic polymer formed by the oxidative coupling of 4-hydroxyphenylpropanoids [12]. The primary lignin precursors are coniferyl, sinapyl, and *p*-coumaryl alcohols (Figure 1). They undergo enzyme-initiated dehydrogenative polymerization during biosynthesis, generating interunit ether and carbon–carbon linkages within the lignin macromolecule [13]. The most predominant linkage is the β-*O*-4 linkage, which comprises approximately 50% of the total linkages [14]. Lignin polymers are considerably more reactive than cellulose or other natural polymers. They lack a repetitive order of units, instead showcasing a diverse array of functional groups, including methoxyl, carboxyl, carbonyl, hydroxyl, and some minor terminal aldehyde groups [15]. The presence and quantities of these functional groups vary depending on the lignin’s origin and extraction process [3].

Lignin is found in higher plant tissues as a cell wall component that provides rigidity and strength, controls water conduction, and protects the plant from microbial degradation [16]. It is one of the most abundant natural terrestrial polymers, with approximately 300 billion Mg on earth [17] and a biosynthetic production rate of 100 million Mg annually [18]. In plant tissue, lignin does not exist as an independent polymer, but it is linked with hemicellulose through covalent bonds, forming carbohydrate polymer matrixes termed lignin–carbohydrate complexes [19].

The plant cell wall is a metabolically active, dynamic compartment with different layers, and each layer has different compositions and attributes [20]. The composition of a typical softwood cell wall includes the primary wall, the secondary wall, which is divided into three sublayers (outer, middle, and inner layer), and the middle lamella. In plant tissues that undergo lignification, such as the sclerenchyma, epidermis (not lignified in legumes), xylem, and non-chlorenchymas parenchyma, lignin synthesis starts during secondary cell wall formation at the cell corners in the middle lamella and the primary wall when the outer layer formation initiates [1,21]. The middle lamella, primary wall as well as the outer layer are mostly composed of lignin (80 and 52.7% of the total weight). However, because the secondary wall occupies a larger portion of the wall, it is recognized for having the highest lignin content [22].

During lignin deposition, monolignols (coniferyl, sinapyl, and *p*-coumaryl alcohols) are synthesized in the cytoplasm from phenylalanine via general phenyl-propanoid and monolignol-specific pathways and transported to the cell wall, where they undergo oxidation and polymerization reactions to form lignin (i.e., lignification). The formation of *p*-hydroxyphenyl (H), guaiacyl (G), and syringyl (S) units occurs when the respective monolignols are incorporated into the lignin polymer [1].

Lignins can be classified into three major groups [20], namely softwood or guaiacyl (formed mostly from G structures), hardwood or guaiacyl–syringyl (formed by a mixture of S and G structures), and grass or syringyl–*p*–hydroxyphenyl lignin (formed from all three monomers). The degree of cross-linking within each lignin group is a crucial factor in determining the rigidity of its structure, which plays a significant role in shaping the overall physicochemical properties of lignin and, consequently, its potential applications [23,24]. For example, lignin from softwood is branched and cross-linked, whereas lignin from hardwood is more linear due to the syringyl unit, which facilitates the retention of the linear structure [25].

## 3. Technical Lignins and Their Antimicrobial Characteristics

**Figure 2:**
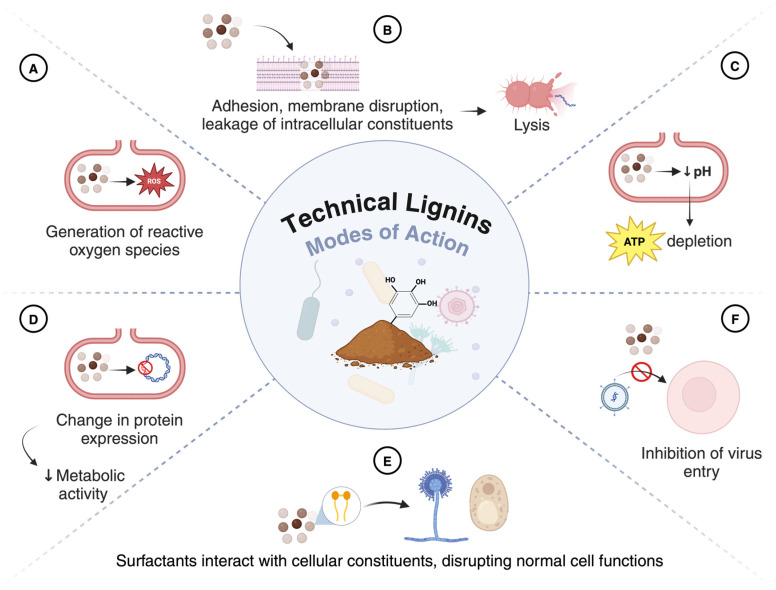
Antimicrobial mechanisms of technical lignins and their derivatives. (**A**) Lignin polyphenols induce oxidative stress within bacterial cells by generating reactive oxygen species (ROS), thereby causing cellular damage [6,28]; (**B**) lignin nanoparticles penetrate bacterial cell walls, disrupting membranes and altering permeability, leading to cell lysis [28,29]; (**C**) the generation of ROS reduces intracellular pH and depletes ATP [28]; (**D**) lignin particles bind with cytoplasmic components, potentially altering or inhibiting the expression of key metabolic proteins [28,29]; (**E**) certain lignin types possess strong surfactant properties that interact with lipids and proteins, adversely affecting fungal growth and viability [30,31]; (**F**) lignin particles interfere with viral entry by interacting with virus envelopes [8].

Among the processes used to extract lignin, Kraft pulping is the most important pulping process globally [26]. Other major processes include sulfite, soda, and organosolv pulping [27]. The extraction and isolation of lignin from lignocellulosic materials are conducted under diverse conditions and multiple reactions (e.g., catalyzed biomass hydrolysis and condensation of lignin fragments), resulting in products with different physicochemical properties [24]. Therefore, in addition to the lignin source, the extraction methods also substantially affect the structure and antimicrobial properties of the technical lignin [5]. Not all types of technical lignins have strong antimicrobial activity. In this review, only the ones with reported antimicrobial properties are discussed. Figure 2 illustrates the proposed antimicrobial modes of action attributed to technical lignins and their derivatives, and Table 1 summarizes the antimicrobial activity tests conducted in the studies.

***Lignosulfonates.*** Lignosulfonates are water-soluble polyelectrolyte polymers that consist of both a hydrophobic aromatic structure and hydrophilic sulfonated groups [61]. They are produced from the sulfite pulping process using sulfur dioxide and a base [5]. The base used is calcium, ammonium, magnesium, or sodium hydroxide typically, and its solubility and dissociation properties influence the pH of the process [24]. Lignosulfonates can be obtained by diverse methods, including alcoholic fermentation followed by distillation, ultrafiltration, or precipitation [5]. The production of lignosulfonates has been reported as 1.8 million tons per year [62], constituting 90% of total commercial lignin. The variety of existing functional groups (hydroxyl-, carboxylic-, and sulfur-containing groups) provides lignosulfonates with distinctive colloidal properties, including superior wettability, dispersive ability, and absorptivity. Consequently, lignosulfonates are commercially used as dispersing agents, binders, adhesives, and stabilizers [62]. Currently they are predominantly used as concrete dispersing agents as well as binders in animal feed pellets and agricultural fertilizers [5].

Lignosulfonates have a broad spectrum of antimicrobial activities. It is hypothesized that the strong surfactant properties of lignosulfonates may explain their antimicrobial activity [30]. Lignosulfonates are considered anionic surfactants due to the presence of the sulfonate groups [61]. The shape and distribution of the charged and uncharged groups in the lignosulfonates’ macromolecular surfaces determines their ability to interact with other molecules [63]. This feature is critical to the antimicrobial activity of lignosulfonates. They can interact with different cellular constituents, especially lipids and proteins, potentially causing adverse effects on the growth and viability of microbial cells by disrupting normal cellular functions [64,65].

Jha and Kumar reported MIC (minimum inhibitory concentration) values for sodium lignosulfonate of 50, 62, 62, 60, and 80 μg/mL for *Candida dubliniensis*, *C. tropicalis*, *C. albicans*, *C. glabrata*, and *C. parasilopsis*, respectively. When these values were evaluated using the disk diffusion method, it was observed that relative to fluconazole (a commonly used antimycotic drug for yeast infections), the inhibition of diameter growth was 6, 10.3, and 23% for *C. glabrata*, *C. tropicalis*, and *C. albicans*, respectively [7]. Similarly, Núñez-Flores et al. reported that an undisclosed dose of sodium lignosulfonate (NaL; 4% reducing sugar content; 7085 Da) extracted from eucalyptus wood showed a 9.9% growth inhibition for *D. hansenii* using the disk diffusion method. However, no antifungal activity was observed against *Aspergillus niger* or *Penicillium expansum* [30]. In another study conducted by Reyes et al., the antifungal activity of NaL and magnesium lignosulfonate (MgL) was evaluated in three subsequent experiments against the molds *Aspergillus amoenus, Mucor circinelloides*, *Penicillium solitum*, and the yeast *Debaromyces hansenii* isolated from spoiled forage [31]. The screening experiment tested lignins at 40 mg/mL using the poisoned food technique. At a pH of 4, NaL was the most effective lignin across fungi (100% inhibition), while for MgL different antifungal activities were observed for *D. hansenii*, *M. circinelloides*, *A. amoenus*, and *P. solitum* (100, 72.9, 40.9, and 28.1%, respectively). The results obtained for NaL by Reyes et al. are comparable to those reported by Jha and Kumar with *Candida* spp. and Núñez-Flores et al. with *D. hansenii* [30,31]. However, Núñez-Flores et al. did not observe an inhibitory activity for NaL against *Penicillium* and *Aspergillus,* while Reyes et al. observed MIC values against *P. solitum* and *A. amoenus* of 33.3 and 20.0 mg/mL, respectively, at pH 4 [30]. This discrepancy may be due to the different *Penicillium* and *Aspergillus* species tested, as well as the NaL sources and doses used across studies. Furthermore, these studies did not report medium pH values. Reyes et al. observed that medium pH plays a major role in the extent of the antifungal activity of technical lignins, with a lower pH (4 vs. 6) resulting in greater inhibition [31]. Furthermore, the authors reported the lowest MIC of NaL across fungi, with values of 20.0, 33.3, 40.0, and 25.0 mg/mL for *A. amoenus*, *P. solitum, M. circinelloides*, and *D. hansenii*, respectively. In the case of MgL, MIC values of 33.3, 46.7, 36.7, and 26.7 mg/mL for each respective fungus were reported at pH 4. Additionally, the authors evaluated the antifungal activity of dose-optimized NaL and MgL on a ground high-moisture alfalfa hay aerobic incubation assay. They found that, at a 3% dose, NaL had superior activity in reducing total mold counts relative to untreated hay (3.92 vs. 7.76 log CFU/fresh g, respectively) [31]. Similarly, León-Tinoco et al. tested the antifungal activity of NaL from four different sources against the same fungi used by Reyes et al. They reported that NaL from Sappi NA (Skowhegan, ME, USA). is the most effective inhibitor and fungicide (except for *M. circinelloides*) at pH 4, with MIC values of 16.0, 15.0, 15.0, and 13.8 mg/mL for *A. amoenus*, *P. solitum*, *M. circinelloides*, and *D. hansenii*, respectively, and MFC (minimal fungicidal concentration) values of 29.0, 31.0, and 13.8 mg/mL for *A. amoenus*, *P. solitum*, and *D. hansenii*, respectively [32].

Regarding antibacterial activity, Kim et al. reported that lignosulfonate nanoparticles engineered from calcium lignosulfonate had a bacteriostatic effect against several bacteria species. At a dose of 5 × 10^10^ particles/mL, the nanoparticles inhibit *Staphylococcus aureus*, *Bacillus subtilis,* and *Escherichia coli* by 95, 58, and 13%, respectively, using a turbidimetric method [11]. Likewise, Reyes et al. evaluated the antibacterial activity of NaL and MgL against strains of *Streptococcus uberis*, *Staphylococcus hyicus*, *E. coli*, *Klebsiella pneumoniae*, and *Pseudomonas aeruginosa* isolated from mastitic and metritic cows [33]. The Gram-positive bacteria (*S. hyicus* and *S. uberis*) were more susceptible to the antimicrobial activity of NaL, with MIC values of 6.25 and 5.8 mg/mL and MBC (minimum bactericidal concentration) values of 8.75 and 5.8 mg/mL, respectively. In Gram-negative bacteria, *E. coli* was the most resistant (MIC and MBC values of 27.5 and 30.0 mg/mL, respectively). Relative to NaL, MgL showed greater inhibition effect against *E. coli* (MIC of 20.0 vs. 27.5 mg/mL) and *K. pneumoniae* (MBC of 40.0 vs. 10.0 mg/mL), respectively.

Furthermore, it is noteworthy to mention that antiviral activity has also been reported for lignosulfonates [34]. Sodium lignosulfonate was highlighted as a potential microbicide with anti-HSV (herpes simplex virus) and anti-HIV (human immunodeficiency virus) activity [8].

***Kraft lignin.*** In the Kraft process, an aqueous solution of sodium hydroxide and sodium sulfide is used to obtain cellulose pulp under a strong alkaline environment in a large pressure vessel or digester, followed by a final acidification process [66]. This digestion causes the lignin polymer to fragment due to the extensive cleavage of β-aryl links and, consequently, the generation of free hydroxyl groups [67]. The Kraft delignification process occurs in three stages at temperatures of 150 °C, between 150 and 170 °C, and >170 °C, respectively. Then, lignin is recovered from the black liquor by decreasing the pH to 5 with sulfuric acid [68]. Kraft lignin is hydrophobic. Therefore, it needs to be modified to improve its solubility [24]. Kraft lignin has a molecular weight ranging from 200 to 200,000 g/mol [34] and an ash content of <3% dry matter [5]. Its production is reported to comprise 95% (47 million Mg) of all lignin produced worldwide [3]. However, Kraft lignin is mostly used in low added-value applications (e.g., power generation) [69], and only about 100,000 Mg per year are commercially used in other products and applications [70]. These include binders and resins [71], carriers for fertilizers and pesticides [72], and the production of low molecular weight compounds, such as vanillin, aliphatic acids, and hydroxylated aromatics [73].

Fewer antimicrobial studies are available for Kraft lignins. Reyes et al. evaluated the antifungal activity of a set of Kraft lignins, including alkali Kraft lignin (AKL), southern pine Kraft lignin (LBKL), LBKL acetone-insoluble (AIF), LBKL acetone soluble/hexane soluble (HEX), and LBKL acetone soluble/hexane insoluble (PI) fractions, at a concentration of 40 mg/mL and pH 4, against *A. amoenus*, *M. circinelloides*, *P. solitum*, and *D. hansenii*, using the poisoned food technique [31]. Across molds, AKL had the highest inhibitory activity (17.9%), followed by PI (12.1%). However, PI was the only Kraft lignin that inhibited the growth of *M. circinelloides* (8.1%). For *D. hansenii*, PI had the highest antifungal activity (10.6%), followed by AKL (9.8), LBKL (8.4), HEX (1.4), and AIF (−2.2) [31]. Similarly, the antimicrobial performance of Kraft lignin extracted from bagasse and cotton stalks against *E. coli*, *Bacillus mycoides*, *B. subtillis*, and *A. niger* was evaluated using a disk diffusion method [35]. While no activity was reported against *E. coli* and *A. niger*, the Kraft lignin showed strong inhibition of the Gram-positive bacteria *B. mycoides* and *B. subtillis*. Likewise, Durmaz et al. evaluated the antifungal activity of Kraft black liquor extracted from Scots pine and reported that a concentration of 5% liquor protected wood samples from fungal degradation by two species of brown-rot fungi, namely *Coniophora puteana*, and *Poria placenta* [36].

When the antimicrobial properties of AKL were evaluated, Dong et al. reported MIC values of 0.01 and 0.0025 μg/mL for *Candida lipolytica* and *S. aureus*, respectively. However, no antibacterial activity was reported against the Gram-positive *Listeria monocytogenes* [6]. Also, Reyes et al. did not observe an MIC for *A. amoenus*, *M. circinelloides*, *P. solitum*, and the yeast *D. hansenii* (>60 mg/mL threshold) [31]. The authors attributed AKL’s lack of activity against the yeast, in contrast to the findings of Dong et al., to differences in the evaluated species, considering that the lignin source and the methodologies were comparable.

Gordobil et al. evaluated the antimicrobial properties of Kraft spruce and Kraft eucalyptus lignins against *A. niger* and 9 pathogenic bacterial strains using a fungal growth inhibition test and a disk diffusion method, respectively [37]. Both lignins showed significant antifungal activity at 1 mg/mL (the lowest concentration tested) with inhibition values of 85 and 76%, respectively. For bacteria, the Kraft spruce lignin had greater antimicrobial activity against *Bacillus thuringiensis*, *E. coli*, *Enterobacter aerogenes*, *Proteus microbilis*, *Proteus vulgaris*, and *S. aureus*, with inhibition zone diameters of 18.4, 17.7, 16.5, 19.1, 24.7, and 19.2 mm, respectively. The Kraft eucalyptus lignin showed higher inhibition against *Streptococcus mutans* and *Salmonella typhimurium*, with inhibition zone diameters of 33.4 and 17.0 mm, respectively [37]. Similarly, Wang et al. evaluated the antibacterial activity of bamboo Kraft lignin (BKL) and BKL 95% ethanol soluble (Fs) and insoluble (Fi) fractions against *S. aureus*, *B. subtilis*, *E. coli*, and *Salmonella enterica* using the agar diffusion and MIC assays [38]. At the concentration tested (20 mg/mL), no obvious difference was observed in the agar diffusion assay between Gram-positive and Gram-negative bacteria. The most effective fraction was Fs, with MIC values of 3, 3, 2, and 2 mg/mL for *E. coli*, *S. enterica*, *B. subtilis*, and *S. aureus*, respectively. This was explained due to a higher phenolic -OH content and water solubility for Fs [38]. Likewise, Yun et al. investigated the antibacterial activity of bamboo Kraft lignin and its acetone, hexane, and non-evaporated fractions against various bacterial strains (*E. coli*, *S. aureus*, and *Streptococcus* and *Salmonella*) using microdilution, agar diffusion, and extracellular protein assays [29]. They observed significant inhibitory effects of all fractions and Kraft lignin on bacterial growth. However, the non-evaporated fraction showed the highest activity, inhibiting growth at a concentration of 0.4 mg/mL. Furthermore, treated bacterial cultures exhibited increased protein content compared to the control, suggesting damage to the bacterial cytoderm. Scanning electron microscope also revealed morphological changes in the bacterial membrane structure upon treatment with the non-evaporated fraction. Similarly, Alzagameem et al. tested the antibacterial activity of four Kraft lignins fractions, extracted subsequently from stirring at pH 2 for 90–180 min, soaking in diethyl ether, acetone, and ethanol. The bacteria tested included *S. aureus*, *L. monocytogenes*, and *E. coli* and the disk diffusion method (inoculum size 10^7^ CFU/mL) was used [39]. The most effective fraction against both *L. monocytogenes* and *S. aureus* was the first fraction (5 and 2 mm, respectively), whereas the diethyl ether and acetone fractions showed inhibitory activity only against *L. monocytogenes* (1 mm). In another study, Reyes et al. evaluated the antibacterial activity of LBKL and AKL against *S. uberis*, *S. hyicus*, *E.coli*, *K. pneumoniae*, and *P. aeruginosa* [33]. They observed MIC values of 16, 10, and 2.5 mg/mL for *S. hyicus*, *S. uberis*, and *K. pneumoniae* for AKL, respectively, while no inhibitory effect was observed against *E. coli* and *P. aeruginosa*. LBKL showed no antimicrobial activity against any bacterium.

The mode of action of Kraft lignins is not clear, and the mechanism against fungi is unknown. For bacteria, Dizhbite et al. suggested that Kraft lignins are associated with the inhibition of radical processes of bacterial cells. Hence, a correlation between radical scavenging (antioxidant) and antimicrobial activities was hypothesized [74]. Similarly, Dong et al. reported a positive association between the antimicrobial and antioxidant activities of lignins [6]. Conversely, other studies did not find such a relationship, as the technical lignins with higher radical scavenging activity were less antifungal [30,31].

***Lignins and their derivatives.*** Lignin is a natural source of phenolic compounds [75]. Phenolic monomers, such as carvacrol and cinnamaldehyde, have shown antimicrobial effects when tested in fresh fruits and vegetables and meat [40,41]. Early studies have reported that lignin constituents (i.e., phenolic monomeric fragments), such as isoeugenol and ferulic acid, can inhibit the growth of *S. cerevisiae*, *C. albicans*, and *A. niger* at doses of 100 and 187, 100 and 375, and 250 and 700 μg/mL, respectively [42]. Similarly, when vanillin, eugenol, and cinnamaldehyde were extracted from lignin by alkaline oxidation with benzene, they were fungicidal at the doses of 0.01% against *Fusarium* spp. [43]. De Greef and van Sumere found that ferulic acid at 2.5 mM had antifungal activity against *S. cerevisiae* [44]. Likewise, Baranowski et al. reported that ferulic acid harbors antimicrobial activity against the same organism at a dose of 0.23 mM [45]. The difference between these two studies was attributed to the lower pH of the medium in the second study (6.0 vs. 3.5, respectively), given that at a lower pH, the efficacy of ferulic acid is boosted due to enhanced membrane permeability in the undissociated state [45].

The antimicrobial activity of the three main classes of intermediates of the lignin-specific pathway (hydroxycinnamaldehydes, hydroxycinnamic acids, and hydroxycinnamyl alcohols) was reported for *S. cerevisiae*, *Schizosaccharomyces pombe*, *Sporobolomyces roseus*, *B. subtilis*, *E. coli*, and *Pseudomonas syringae* [46]. Hydroxycinnamaldehydes were the strongest antimicrobial compounds, with coniferaldehyde being the most antifungal (MIC 1.2 mM) and *p*-coumaraldehyde being the most antibacterial (MIC 2.0 mM). In the case of the hydroxycinnamic acids (*p*-coumaric, cafeic, ferulic, and sinapic acid), a higher inhibitory effect against bacteria relative to fungi was observed (MIC 3.0 vs. >8 mM), except for ferulic acid, which inhibits *S. cerevisiae* at a concentration of 4.0 mM. The hydroxycinnamyl alcohols (*p*-coumaryl, coniferyl, and sinapyl alcohol) had the lowest antimicrobial properties (MIC ≥ 8.0 mM) [46].

Lignin–carbohydrate complexes (LCCs) are hybrid structures composed of covalently linked lignin and carbohydrate moieties, chemically bound in native biomass, that play a crucial role in wood structure [76]. LCCs have been reported to have antimicrobial, antiparasitic, antitumor, and antiviral properties [54,77,78,79]. An LCC extracted from pine trees had an inhibitory effect against *S. aureus*, *E. coli*, *Pseudomonas aeruginosa*, *Klebsiella pneumoniae*, and *C. albicans*, but no antibacterial activity was reported against *Salmonella enteriditis* in mice at an undisclosed dose [51,52]. It was suggested that the sugar moiety of LCC had a significant influence on the induction of antimicrobial activity because when the sugar fraction was removed with sulfuric acid, the antimicrobial activity decreased significantly [77]. Moreover, LCC extracted from a pine species (*Pinus parviflora*) with an alkaline solution, evaluated at an undisclosed dose, showed a high anti-tumor activity in mice, which was increased when LCC was acidified [77]. In addition, the same LCC extract applied subcutaneously at a dose of 10 mg/kg live weight protected mice from infection caused by the cestode *Hymenolepis nana*. Moreover, LCCs extracted from pinecone [53], *Theobroma cacao* [54], and mulberry juice [80] showed unique antiviral activity in cell lines against HIV, HSV, and influenza viruses.

***Soda lignin.*** This type of lignin is produced by treating non-wood fibers, such as bagasse, flax, straws, or sugarcane, with highly alkaline solutions of sodium hydroxide, and unlike Kraft lignin, the cooking process is performed in a sulfur-free medium [81]. The chemical properties of the soda lignin are considerably different from lignosulfonates, as these are hydrophobic lignins with lower molecular weights (ranging from 1000 to 3000 g/mol) [5]. Due to the absence of sulfur, it is suggested that the composition of soda lignin is closer to native lignin relative to other technical lignins [82]. Potential applications in certain areas, such as animal feed and nutrition, have been reported, particularly for the treatment of enteric disturbances in ruminants [83], and as alternatives for antibiotics [5].

Very few studies have investigated the antimicrobial properties of soda lignin. One study reported that soda lignin extracted from sugarcane bagasse had MIC and MBC values against *Staphylococcus epidermidis* at 4096 and 8192 µg/mL, respectively [47]. When this soda lignin was used to coat fabric at the MBC concentration, further bacterial growth was inhibited [47]. In another study, technical lignin from Bagasse obtained by soda treatment cooking at 130 °C had significant antimicrobial activity against *Bacillus* spp., but not *E. coli* or *A. niger.* In addition, when the cooking temperature was raised to 160 °C, no antimicrobial activity was observed, indicating that even under the same type of treatment, the temperature difference can cause antimicrobial property variation in end products [35].

***Organosolv lignin.*** The organosolv process includes the solubilization of wood using a mixture of organic solvents, predominantly formic or acetic acid, and ethanol, followed by filtration and drying [84]. Organosolv lignin has a high lignin purity due to its minimal carbohydrate and ash content [24]. It is hydrophobic and has a low molecular weight (500 to 5000 mol/g) [23]. Several organosolv pulping processes are commercially registered, among which organosolv lignin from the Alcell (extracted with ethanol) process has been the most studied to date [4]. Alcell lignin has been reported to have in vitro [48,85] and in vivo antibacterial activity [49], as well as prebiotic effects, including improving intestinal morphology and supporting the growth of beneficial bacteria in broiler chickens [86]. Furthermore, Wang et al. reported linear reductions in methane emissions and ammonia-N accumulation in 24 h when Alcell lignin was added to feedlot lamb diets and fermented in vitro [87], indicating enhanced energy utilization efficiency [88] and reduced environmental pollution [89]. In addition, one study showed that the n-hexane soluble fraction of organosolv lignin can inhibit the growth of *Trametes versicolor* (white rot fungi), and with chemical modification analysis, it was confirmed that the phenolic hydroxyl group was responsible for the antifungal activity [50].

***Lignin-based biopolymers.*** The risk of bacterial colonization and fungal contamination is a frequent complication associated with the use of biomedical devices. The exploration of lignin’s antibacterial and antifungal properties led to the demand for novel lignin-based biopolymers (films, fibers, and hydrogels) and lignin nanoparticles (LNP). Larrañeta et al. investigated the potential of lignin-based hydrogels for biomedical applications as coating materials. Substantial superior resistance to bacterial adherence from hydrogels containing 38% (*w*/*w*) dealkaline lignin relative to a commonly employed medical material was observed against *S. aureus* and *Proteus mirabilis* [10]. In the same study, lignin-based hydrogels were evaluated as hydrophobic drug delivery systems, and it concluded that those same hydrogels were able to sustain the release of curcumin for up to 4 d [10]. Moreover, nanocomposite fibers made from alkali Kraft lignin with low sulfonate content were tested against *S. aureus* and *E. coli*. Fibers with 29% (*w*/*w*) lignin showed a 99.9% reduction rate in *S. aureus* populations. However, no inhibition was observed for *E. coli* [55]. Regarding nanotechnology development, LNPs synthesized by dissolving alkali Kraft lignin into ethylene glycol followed by acidolysis were tested against plant pathogens including *Pseudomonas syringae*, *Xanthomonas axonopodis*, and *Xanthomonas arboricola* [28]. The LNP showed effective antimicrobial activity against all these pathogens. Especially at a dose of 4%, LNP had the highest antibacterial activity against *X. arboricola* with a 3-log reduction (1 × 10^8^ to 5 × 10^4^ CFU/mL) after 24 h of incubation using a broth susceptibility assay. These results indicate that the LNPs tested have great potential to protect plants from these pathogens and reduce the heavy economic losses on plum, peach, apricot, and cherry trees. The authors suggested that there are two possible antimicrobial mechanisms of LNPs [28]. First, lignin polyphenols cause cell wall damage and bacterial cell internal fluid leakage by inducing oxidative stress. This theory assumes that the accumulation of reactive oxygen species (ROS) absorbed by the polyphenol compounds occurs on the surface of LNP. Another theory is that, because of their small size, LNPs can penetrate the bacterial cell, evading the cell membrane, decrease the intracellular pH and, consequently, ATP levels, and then lead to the cell’s death.

***Non-conventional lignins.*** Lignocellulosic materials, such as crop residues, are abundant, readily available, and low-cost [24]. The antibacterial properties of lignins extracted from sugarcane bagasse with alkaline solutions and different modified lignins i.e., acetylated, epoxy, and hydroxymethyl lignin, were evaluated against *Bacillus aryabhattai* and *Klebsiella* spp. using the disk diffusion method [56]. Epoxy lignin (lignin extracted using a chlorinated cyclic ether) was the most effective among unmodified and modified lignins, with MIC values of 90 and 200 µg/disk for each bacterium, respectively. The presence of methoxyl and epoxy groups in lignin was responsible for the enhanced antibacterial activity of the lignin modified by epoxidation [56]. Similarly, the antifungal properties of different lignin fractions from apple tree pruning waste obtained by autohydrolysis, organosolv treatment with acid or ethanol and soda hydrolysis were investigated against *A. niger*, and *S. cerevisiae* [57]. None of the lignins tested exhibited antifungal activity against *A. niger* for any lignin at doses of 500 and 5000 ppm, and in fact, the lower dose enhanced mold growth, which was explained by the presence of minerals and hemicelluloses of the lignin fractions [57]. However, all of the tested lignin fractions decreased the growth of *S. cerevisiae* at 5000 and 10,000 ppm, with autohydrolysis lignin at 10,000 ppm being the most antifungal fraction, as shown by a 78.7% decrease in growth vs. control using a spectrophotometric method. In addition, the pigmentation of *A. niger* was affected by lignin fractions at doses of 5000 ppm, with the colonies exhibiting pale blue, green, or yellow pigmentation, compared to colonies growing on the control plates [57,79]. These results agree with Rahouti et al., who studied seven phenolic lignin model compounds against various fungi and observed that guaiacol and syringic acid induce the production of atypical pigments and viscous compounds [58]. Likewise, Coral Medina et al. evaluated the antimicrobial activity of one lignin isolated from oil palm empty fruit bunches using a sequential acid–alkaline pretreatment [59]. The lignin tested was selected for having the highest total phenolic content (181.21 mg GAE/mg) among six lignin candidates. However, this lignin did not have an effect against *C. albicans* or *A. niger* at 2000 µg/mL using the disk diffusion method. Conversely, using a broth antimicrobial assay, the lignin exhibited antibacterial activity, with the greatest inhibition observed at 250 µg/mL for *B. subtilis* (39%), 1000 µg/mL for *S. enterica* (31%), and 2000 µg/mL for *E. coli* (50%), and *S. aureus* (67%) [59].

## 4. Conclusions

The interest in naturally derived antimicrobial compounds is constantly developing, with particular attention being directed towards technical lignins, which exhibit promising antimicrobial properties. These lignins, modified into various forms, have garnered interest for potential application in fields ranging from agriculture and biomedicine to food science and pharmacology. However, the diverse nature of technical lignins poses a challenge in conclusively determining their antimicrobial properties. With many different types of technical lignin available, each with its own chemical composition and characteristics, it becomes difficult to establish consistent findings across studies. Compounding this challenge is the lack of standardized antimicrobial assays and units used in research, further complicating the comparison and interpretation of results. Consequently, efforts to elucidate the antimicrobial characteristics of technical lignins are hindered by these inconsistencies and gaps in data. Nonetheless, recent studies have contributed to a deeper understanding of technical lignins’ antimicrobial activity, shedding light on their potential as valuable resources in combating microbial threats across diverse industries.

## Figures and Tables

**Table 1:** Summary of the antimicrobial activity tests conducted in cited studies.

Technical Lignin	Pathogens Tested	Antimicrobial Test Method	Reference
Sodium lignosulfonate	*Candida dubliniensis* *C. tropicalis* *C. albicans* *C. glabrata* *C. parasilopsis*	MIC ^1^; Disk diffusion assay	[7]
Sodium lignosulfonate	*D. hansenii* *Aspergillus niger* *Penicillium expansum*	Disk diffusion assay	[30]
Sodium lignosulfonate; magnesium lignosulfonate; alkali kraft lignin; southern pine kraft lignin (LBKL); LBKL acetone-insoluble;	*Aspergillus amoenus* *Mucor circinelloides* *Penicillium solitum* *Debaromyces hansenii*	Broth antimicrobial assay; MIC at different pH levels	[31]
Sodium lignosulfonate	*A. amoenus* *M. circinelloides* *P. solitum* *D. hansenii*	MIC and MFC ^2^	[32]
Lignosulfonate nanoparticles	*Staphylococcus aureus* *Bacillus subtilis* *Escherichia coli*	Turbidimetric method	[11]
Sodium lignosulfonate; magnesium lignosulfonate; alkali kraft lignin; LBKL	*Streptococcus uberis* *Staphylococcus hyicus* *E. coli* *Klebsiella pneumoniae* *Pseudomonas aeruginosa*	MIC and MBC ^3^	[33]
Lignosulfonate	HIV ^4^	Virus antigen expression; cytopathic effect evaluation; cell-to-cell infection; reverse transcriptase assay	[34]
Lignosulfonic acid	HIV HSV ^5^	Virus replication assay; virus time-of-drug-addition assay; virus inactivation assay; in vivo antiviral activity in mice	[8]
Kraft lignins; soda lignins	*E. coli* *Bacillus mycoides* *B. subtillis* *A. niger*	Disk diffusion assay	[35]
Kraft black liquor	*Coniophora puteana* *Poria placenta*	Wood protection from fungal degradation	[36]
Alkali kraft lignin	*Candida lipolytica* *S. aureus* *Listeria monocytogenes*	MIC	[6]
Kraft spruce lignins; Kraft eucalyptus lignins	*A. niger* *B. thuringiensis* *E. coli* *Enterobacter aerogenes* *Proteus microbilis* *P. vulgaris* *S. aureus*	Fungal growth inhibition test; disk diffusion assay	[37]
Bamboo kraft lignin (BKL); BKL 95% ethanol soluble fraction; BKL 95% ethanol insoluble fraction	*S. aureus* *B. subtilis* *E. coli* *Salmonella enterica*	MIC; agar diffusion assay	[38]
BKL; BKL acetone fraction; BKL hexane fraction; BKL non-evaporated fraction	*E. coli* *S. aureus* *Streptococcus pyogenes* *S. enterica*	Agar diffusion assay; microdilution assay; extracellular protein assay; in vivo antimicrobial activity with mice	[29]
Kraft lignin fractions	*S. aureus* *L. monocytogenes* *E. coli*	Disk diffusion assay	[39]
Carvacrol	*B. cereus*	Antimicrobial activity in rice	[40]
Carvacrol; cinnamaldehyde	Spoilage microbial flora	Antimicrobial activity in melon and kiwifruit	[41]
Isoeugenol; ferulic acid	*S. cerevisiae* *C. albicans* *A. niger*	MIC	[42]
Vanillin; eugenol; cinnamaldehyde	*Fusarium* spp.	Antifungal activity	[43]
Ferulic acid	*S. cerevisiae*	Antimicrobial activity	[44]
Ferulic acid	*S. cerevisiae*	Antimicrobial activity	[45]
Hydroxycinnamaldehydes; hydroxycinnamic acids; hydroxycinnamyl alcohols	*S. cerevisiae* *Schizosaccharomyces pombe* *Sporobolomyces roseus* *B. subtilis* *E. coli* *Pseudomonas syringae*	MIC	[46]
Soda lignin	*Staphylococcus epidermidis*	MIC and MBC	[47]
Alcell lignin	Gut microflora	Antimicrobial activity in vitro; Antimicrobial activity in mice	[48]
Alcell lignin	*E. coli* *Lactobacilli* *Bifidobacteria*	Antimicrobial activities in broilers	[49]
n-hexane-soluble fraction of organosolv lignin	*Trametes versicolor* (white rot fungi)	Disk diffusion assay	[50]
Lignin–carbohydrate complexes	*E. coli*	Antimicrobial activity in mice	[51]
Lignin–carbohydrate complexes	*S. aureus* *E. coli* *P. aeruginosa* *K. pneumoniae* *C. albicans* *S. enteriditis*	Antimicrobial activity in mice	[52]
Lignin–carbohydrate complexes	*HIV*	Antiviral assay in cell lines	[53]
Lignin–carbohydrate complexes	*HIV*	Antiviral assay in cell lines	[54]
Lignin-based hydrogels	*S. aureus* *Proteus mirabilis*	Bacterial adherence resistance	[10]
Kraft lignin nanocomposite fibers	*S. aureus* *E. coli*	ASTM E 2149-10 ^6^	[55]
Kraft lignin nanoparticles	*Pseudomonas syringae* *Xanthomonas axonopodis* *Xanthomonas arboricola*	Spot diffusion assay; bacterial growth in broth	[28]
Acetylated, epoxy, and hydroxymethyl lignin	*Bacillus aryabhattai* *Klebsiella* spp.	Disk diffusion assay; MIC	[56]
Technical lignins obtained by autohydrolysis, organosolv treatment with acid, or ethanol, and soda hydrolysis	*A. niger* *S. cerevisiae*	Broth antifungal assay	[57]
Phenolic lignin compounds	1044 strains and species of fungi	Growth inhibition assay	[58]
Lignin extracted with a sequential acid–alkaline pretreatment	*C. albicans* *A. niger* *B. subtilis* *E. coli* *S. aureus*	Disk diffusion assay Broth antimicrobial assay	[59]

^1^ MIC: minimal inhibitory test. ^2^ MFC: minimal fungicidal concentration. ^3^ MBC: minimal bactericidal concentration. ^4^ HIV: human immunodeficiency virus. ^5^ HSV: herpes simplex virus. ^6^ ASTM E 2149-10: standard test method for determining the antimicrobial activity of immobilized antimicrobial agents under dynamic contact conditions [60].

## References

[1] Cazacu G., Capraru M., Popa V.I., Thomas S., Visakh P.M., Mathew A.P. (2013). Advances Concerning Lignin Utilization in New Materials. Advances in Natural Polymers.

[2] Laurichesse S., Avérous L. (2014). Chemical Modification of Lignins: Towards Biobased Polymers. Prog. Polym. Sci..

[3] Gosselink R.J.A., De Jong E., Guran B., Abächerli A. (2004). Co-Ordination Network for Lignin—Standardisation, Production and Applications Adapted to Market Requirements (EUROLIGNIN). Ind. Crops Prod..

[4] Berlin A., Balakshin M. (2014). Industrial Lignins. Bioenergy Research: Advances and Applications.

[5] Lora J. (2008). Industrial Commercial Lignins: Sources, Properties and Applications. Monomers, Polymers and Composites from Renewable Resources.

[6] Dong X., Dong M., Lu Y., Turley A., Jin T., Wu C. (2011). Antimicrobial and Antioxidant Activities of Lignin from Residue of Corn Stover to Ethanol Production. Ind. Crops Prod..

[7] Jha A., Kumar A. (2018). Deciphering the Role of Sodium Lignosulfonate against *Candida* spp. as Persuasive Anticandidal Agent. Int. J. Biol. Macromol..

[8] Gordts S.C., Férir G., D’huys T., Petrova M.I., Lebeer S., Snoeck R., Andrei G., Schols D. (2015). The Low-Cost Compound Lignosulfonic Acid (LA) Exhibits Broad-Spectrum Anti-HIV and Anti-HSV Activity and Has Potential for Microbicidal Applications. PLoS ONE.

[9] Flickinger E.A., Campbell J.M., Schmitt L.G., Fahey G.C. (1998). Selected Lignosulfonate Fractions Affect Growth Performance, Digestibility, and Cecal and Colonic Properties in Rats. J. Anim. Sci..

[10] Larrañeta E., Imízcoz M., Toh J.X., Irwin N.J., Ripolin A., Perminova A., Domínguez-Robles J., Rodríguez A., Donnelly R.F. (2018). Synthesis and Characterization of Lignin Hydrogels for Potential Applications as Drug Eluting Antimicrobial Coatings for Medical Materials. ACS Sustain. Chem. Eng..

[11] Kim S., Fernandes M.M., Matamá T., Loureiro A., Gomes A.C., Cavaco-Paulo A. (2013). Chitosan–Lignosulfonates Sono-Chemically Prepared Nanoparticles: Characterisation and Potential Applications. Colloids Surf. B Biointerfaces.

[12] Ralph J., Lundquist K., Brunow G., Lu F., Kim H., Schatz P.F., Marita J.M., Hatfield R.D., Ralph S.A., Christensen J.H. (2004). Lignins: Natural Polymers from Oxidative Coupling of 4-Hydroxyphenyl-Propanoids. Phytochem. Rev..

[13] Kai D., Tan M.J., Chee P.L., Chua Y.K., Yap Y.L., Loh X.J. (2016). Towards Lignin-Based Functional Materials in a Sustainable World. Green Chem..

[14] Hatfield R., Vermerris W. (2001). Lignin Formation in Plants. The Dilemma of Linkage Specificity. Plant Physiol..

[15] Adler E. (1977). Lignin Chemistry? Past, Present and Future. Wood Sci. Technol..

[16] Boerjan W., Ralph J., Baucher M. (2003). Lignin Biosynthesis. Annu. Rev. Plant Biol..

[17] Argyropoulos D.S., Menachem S.B., Eriksson K.-E.L., Babel W., Blanch H.W., Cooney C.L., Enfors S.-O., Eriksson K.-E.L., Fiechter A., Klibanov A.M., Mattiasson B., Primrose S.B. (1997). Lignin. Biotechnology in the Pulp and Paper Industry.

[18] Bajwa D.S., Pourhashem G., Ullah A.H., Bajwa S.G. (2019). A Concise Review of Current Lignin Production, Applications, Products and Their Environmental Impact. Ind. Crops Prod..

[19] Ralph J., Grabber J.H., Hatfield R.D. (1995). Lignin-Ferulate Cross-Links in Grasses: Active Incorporation of Ferulate Polysaccharide Esters into Ryegrass Lignins. Carbohydr. Res..

[20] Evert R.F. (2006). Esau’s Plant Anatomy: Meristems, Cells, and Tissues of the Plant Body: Their Structure, Function, and Development.

[21] Terashima N., Fukushima K., He L.-F., Takabe K., Jung H.G., Buxton D.R., Hatfield R.D., Ralph J. (2015). Comprehensive Model of the Lignified Plant Cell Wall. ASA, CSSA, and SSSA Books.

[22] Baucher M., Monties B., Montagu M.V., Boerjan W. (1998). Biosynthesis and Genetic Engineering of Lignin. Crit. Rev. Plant Sci..

[23] Cadix A., James S. (2022). Cementing Additives. Fluid Chemistry, Drilling and Completion.

[24] Doherty W.O.S., Mousavioun P., Fellows C.M. (2011). Value-Adding to Cellulosic Ethanol: Lignin Polymers. Ind. Crops Prod..

[25] Thakur V.K., Thakur M.K., Raghavan P., Kessler M.R. (2014). Progress in Green Polymer Composites from Lignin for Multifunctional Applications: A Review. ACS Sustain. Chem. Eng..

[26] Smook G., Kocurek M. (1982). Handbook for Pulp & Paper Technologists.

[27] Espinoza-Acosta J.L., Torres-Chávez P.I., Ramírez-Wong B., López-Saiz C.M., Montaño-Leyva B. (2016). Antioxidant, Antimicrobial, and Antimutagenic Properties of Technical Lignins and Their Applications. BioRes.

[28] Yang W., Fortunati E., Gao D., Balestra G.M., Giovanale G., He X., Torre L., Kenny J.M., Puglia D. (2018). Valorization of Acid Isolated High Yield Lignin Nanoparticles as Innovative Antioxidant/Antimicrobial Organic Materials. ACS Sustain. Chem. Eng..

[29] Yun J., Wei L., Li W., Gong D., Qin H., Feng X., Li G., Ling Z., Wang P., Yin B. (2021). Isolating High Antimicrobial Ability Lignin From Bamboo Kraft Lignin by Organosolv Fractionation. Front. Bioeng. Biotechnol..

[30] Núñez-Flores R., Giménez B., Fernández-Martín F., López-Caballero M.E., Montero M.P., Gómez-Guillén M.C. (2012). Role of Lignosulphonate in Properties of Fish Gelatin Films. Food Hydrocoll..

[31] Reyes D.C., Annis S.L., Rivera S.A., Leon-Tinoco A.Y., Wu C., Perkins L.B., Perry J.J., Ma Z.X., Knight C.W., Castillo M.S. (2020). In Vitro Screening of Technical Lignins to Determine Their Potential as Hay Preservatives. J. Dairy Sci..

[32] Leon-Tinoco A.Y., Annis S.L., Almeida S.T., Guimarães B.C., Killerby M., Zhang J., Wu C., Perkins L.B., Ma Z., Jeong K.C. (2022). Evaluating the Potential of Lignosulfonates and Chitosans as Alfalfa Hay Preservatives Using in Vitro Techniques. J. Anim. Sci..

[33] Reyes D.C., Rivera S.A., Ma Z.X., Dubuc H.M., Leon-Tinoco A.Y., Lichtenwalner A.B., Romero J.J. (2019). Mitigating Environmental Mastitis Microbes with the Novel Use of Paper Mill Byproducts. J. Dairy Sci..

[34] Suzuki H., Tochikura T.S., Iiyama K., Yamazaki S., Yamamoto N., Toda S. (1989). Lignosulfonate, a Water-Solubilized Lignin from the Waste Liquor of the Pulping Process, Inhibits the Infectivity and Cytopathic Effects of Human Immunodeficiency Virus in Vitro. Agric. Biol. Chem..

[35] Nada A.M.A., El-Diwany A.I., Elshafei A.M. (1989). Infrared and Antimicrobial Studies on Different Lignins. Acta Biotechnol..

[36] Durmaz S., Erisir E., Yildiz U.C., Kurtulus O.C. (2015). Using Kraft Black Liquor as a Wood Preservative. Procedia Soc. Behav. Sci..

[37] Gordobil O., Herrera R., Yahyaoui M., İlk S., Kaya M., Labidi J. (2018). Potential Use of Kraft and Organosolv Lignins as a Natural Additive for Healthcare Products. RSC Adv..

[38] Wang G., Pang T., Xia Y., Liu X., Li S., Parvez A.M., Kong F., Si C. (2019). Subdivision of Bamboo Kraft Lignin by One-Step Ethanol Fractionation to Enhance Its Water-Solubility and Antibacterial Performance. Int. J. Biol. Macromol..

[39] Alzagameem A., Klein S.E., Bergs M., Do X.T., Korte I., Dohlen S., Hüwe C., Kreyenschmidt J., Kamm B., Larkins M. (2019). Antimicrobial Activity of Lignin and Lignin-Derived Cellulose and Chitosan Composites Against Selected Pathogenic and Spoilage Microorganisms. Polymers.

[40] Ultee A., Slump R.A., Steging G., Smid E.J. (2000). Antimicrobial Activity of Carvacrol toward Bacillus Cereus on Rice. J. Food Prot..

[41] Roller S., Seedhar P. (2002). Carvacrol and Cinnamic Acid Inhibit Microbial Growth in Fresh-Cut Melon and Kiwifruit at 4° and 8 °C. Lett. Appl. Microbiol..

[42] Zemek J., Košíková B., Augustín J., Joniak D. (1979). Antibiotic Properties of Lignin Components. Folia Microbiol..

[43] Telysheva G., Sergeeva V., Gavare L. (1968). Fungicidal Properties of Alkali Oxidation Destruction Products of Lignin. Latv. PSR Zinat. Akad. Vestis. Kim. Ser..

[44] De Greef J., Van Sumere C. (1966). Effect of Phenolic Aldehydes, Coumarins and Related Compounds on the Growth of Saccharomyces Cerevisiae. Arch. Int. Physiol. Biochem..

[45] Baranowski J.D., Davidson P.M., Nagel C.W., Branen A.L. (1980). Inhibition of Saccharomyces Cerevisiae by Naturally Occurring Hydroxycinnamates. J. Food Sci..

[46] Barber M.S., McConnell V.S., DeCaux B.S. (2000). Antimicrobial Intermediates of the General Phenylpropanoid and Lignin Specific Pathways. Phytochemistry.

[47] Sunthornvarabhas J., Liengprayoon S., Suwonsichon T. (2017). Antimicrobial Kinetic Activities of Lignin from Sugarcane Bagasse for Textile Product. Ind. Crops Prod..

[48] Nelson J.L., Alexander J.W., Gianotti L., Chalk C.L., Pyles T. (1994). Influence of Dietary Fiber on Microbial Growth in Vitro and Bacterial Translocation after Burn Injury in Mice. Nutrition.

[49] Baurhoo B., Letellier A., Zhao X., Ruiz-Feria C.A. (2007). Cecal Populations of Lactobacilli and Bifidobacteria and Escherichia Coli Populations After In Vivo Escherichia Coli Challenge in Birds Fed Diets with Purified Lignin or Mannanoligosaccharides. Poult. Sci..

[50] Ishimaru H., Umezawa T., Yoshikawa T., Koyama Y., Fumoto E., Sato S., Nakasaka Y., Masuda T. (2023). Antifungal Activity of Simply Fractionated Organosolv Lignin against Trametes Versicolor. J. Biotechnol..

[51] Harada H., Sakagami H., Konno K., Sato T., Osawa N., Fujimaki M., Komatsu N. (1988). Induction of Antimicrobial Activity by Antitumor Substances from Pine Cone Extract of Pinus Parviflora Sieb. et Zucc. Anticancer. Res..

[52] Oh-Hara T., Sakagami H., Kawazoe Y., Kaiya T., Komatsu N., Ohsawa N., Fujimaki M., Tanuma S., Konno K. (1990). Antimicrobial Spectrum of Lignin-Related Pine Cone Extracts of Pinus Parviflora Sieb. et Zucc. Vivo.

[53] Lai P.K., Donovan J., Takayama H., Sakagami H., Tanaka A., Konno K., Nonoyama M. (1990). Modification of Human Immunodeficiency Viral Replication by Pine Cone Extracts. AIDS Res. Hum. Retroviruses.

[54] Sakagami H., Satoh K., Fukamachi H., Ikarashi T., Shimizu A., Yano K., Kanamoto T., Terakubo S., Nakashima H., Hasegawa H. (2008). Anti-HIV and Vitamin C-Synergized Radical Scavenging Activity of Cacao Husk Lignin Fractions. Vivo.

[55] Lee E., Song Y., Lee S. (2019). Crosslinking of Lignin/Poly(Vinyl Alcohol) Nanocomposite Fiber Webs and Their Antimicrobial and Ultraviolet-Protective Properties. Text. Res. J..

[56] Kaur R., Uppal S.K., Sharma P. (2017). Antioxidant and Antibacterial Activities of Sugarcane Bagasse Lignin and Chemically Modified Lignins. Sugar Tech.

[57] García A., Spigno G., Labidi J. (2017). Antioxidant and Biocide Behaviour of Lignin Fractions from Apple Tree Pruning Residues. Ind. Crops Prod..

[58] Rahouti M., Steiman R., Seigle-Murandi F., Christov L.P. (1999). Growth of 1044 Strains and Species of Fungi on 7 Phenolic Lignin Model Compounds. Chemosphere.

[59] Coral Medina J.D., Woiciechowski A.L., Zandona Filho A., Bissoqui L., Noseda M.D., De Souza Vandenberghe L.P., Zawadzki S.F., Soccol C.R. (2016). Biological Activities and Thermal Behavior of Lignin from Oil Palm Empty Fruit Bunches as Potential Source of Chemicals of Added Value. Ind. Crops Prod..

[60] (2020). Standard Test Method for Determining the Antimicrobial Activity of Immobilized Antimicrobial Agents under Dynamic Contact Conditions.

[61] Xu C., Ferdosian F. (2017). Conversion of Lignin into Bio-Based Chemicals and Materials.

[62] Gonçalves S., Ferra J., Paiva N., Martins J., Carvalho L.H., Magalhães F.D. (2021). Lignosulphonates as an Alternative to Non-Renewable Binders in Wood-Based Materials. Polymers.

[63] Vainio U., Lauten R.A., Haas S., Svedström K., Veiga L.S.I., Hoell A., Serimaa R. (2012). Distribution of Counterions around Lignosulfonate Macromolecules in Different Polar Solvent Mixtures. Langmuir.

[64] Merianos J. (1991). Quaternary Ammonium Antimicrobial Compounds. J Disinfect..

[65] Russell A.D., Hugo W.B. (1982). Principles and Practice of Disinfection, Preservation and Sterilisation.

[66] Lange H., Decina S., Crestini C. (2013). Oxidative Upgrade of Lignin—Recent Routes Reviewed. Eur. Polym. J..

[67] Vishtal A., Kraslawski A. (2011). Challenges in Industrial Applications of Technical Lignins. BioRes.

[68] Koljonen K., Österberg M., Kleen M., Fuhrmann A., Stenius P. (2004). Precipitation of Lignin and Extractives on Kraft Pulp: Effect on Surface Chemistry, Surface Morphology and Paper Strength. Cellulose.

[69] Mohan D., Pittman C.U., Steele P.H. (2006). Single, Binary and Multi-Component Adsorption of Copper and Cadmium from Aqueous Solutions on Kraft Lignin—A Biosorbent. J. Colloid Interface Sci..

[70] Mansouri N.-E.E., Salvadó J. (2006). Structural Characterization of Technical Lignins for the Production of Adhesives: Application to Lignosulfonate, Kraft, Soda-Anthraquinone, Organosolv and Ethanol Process Lignins. Ind. Crops Prod..

[71] Tejado A., Peña C., Labidi J., Echeverria J.M., Mondragon I. (2007). Physico-Chemical Characterization of Lignins from Different Sources for Use in Phenol–Formaldehyde Resin Synthesis. Bioresour. Technol..

[72] Zhang B., Huang H.-J., Ramaswamy S. (2008). Reaction Kinetics of the Hydrothermal Treatment of Lignin. Appl. Biochem. Biotechnol..

[73] Holladay J.E., White J.F., Bozell J.J., Johnson D. (2007). Top Value-Added Chemicals from Biomass-Volume II—Results of Screening for Potential Candidates from Biorefinery Lignin.

[74] Dizhbite T. (2004). Characterization of the Radical Scavenging Activity of Lignins–Natural Antioxidants. Bioresour. Technol..

[75] Baurhoo B., Ruiz-Feria C.A., Zhao X. (2008). Purified Lignin: Nutritional and Health Impacts on Farm Animals—A Review. Anim. Feed Sci. Technol..

[76] Balakshin M., Capanema E., Berlin A. (2014). Isolation and Analysis of Lignin–Carbohydrate Complexes Preparations with Traditional and Advanced Methods: A Review. In Stud. Nat. Prod. Chem..

[77] Sakagami H., Kushida T., Oizumi T., Nakashima H., Makino T. (2010). Distribution of Lignin–Carbohydrate Complex in Plant Kingdom and Its Functionality as Alternative Medicine. Pharmacol. Ther..

[78] Abe M., Okamoto K., Konno K., Sakagami H. (1989). Induction of Antiparasite Activity by Pine Cone Lignin-Related Substances. Vivo.

[79] Lee H., Aoki K., Sakagami H., Yoshida T., Kuroiwa Y. (1993). Interaction of Pine Cone Extract Fraction VI with Mutagens. Mutat. Res./Rev. Genet. Toxicol..

[80] Sakagami H., Watanabe S. (2011). Beneficial Effects of Mulberry on Human Health. Phytotherapeutics and Human Health: Pharmacological and Molecular Aspects.

[81] Duval A., Lawoko M. (2014). A Review on Lignin-Based Polymeric, Micro- and Nano-Structured Materials. React. Funct. Polym..

[82] Nadif A., Hunkeler D., Kauper P. (2002). Sulfur-Free Lignins from Alkaline Pulping Tested in Mortar for Use as Mortar Additives. Bioresour. Technol..

[83] Cruz R., Dopico D., Figueredo J., Rodriguez M., Martinez G. (1997). Uso de La Lignina de Bagazo Con Fines Medicinales. Rev. Peru. De Med. Exp. Y Salud Publica.

[84] Xu F., Sun J.-X., Sun R., Fowler P., Baird M.S. (2006). Comparative Study of Organosolv Lignins from Wheat Straw. Ind. Crops Prod..

[85] Ayyachamy M., Cliffe F.E., Coyne J.M., Collier J., Tuohy M.G. (2013). Lignin: Untapped Biopolymers in Biomass Conversion Technologies. Biomass Conv. Bioref..

[86] Baurhoo B., Phillip L., Ruiz-Feria C.A. (2007). Effects of Purified Lignin and Mannan Oligosaccharides on Intestinal Integrity and Microbial Populations in the Ceca and Litter of Broiler Chickens. Poult. Sci..

[87] Wang Y., Marx T., Lora J., Phillip L.E., McAllister T.A. (2009). Effects of Purified Lignin on in Vitro Ruminal Fermentation and Growth Performance, Carcass Traits and Fecal Shedding of Escherichia Coli by Feedlot Lambs. Anim. Feed Sci. Technol..

[88] Ørskov E.R., Flatt W.P., Moe P.W. (1968). Fermentation Balance Approach to Estimate Extent of Fermentation and Efficiency of Volatile Fatty Acid Formation in Ruminants. J. Dairy Sci..

[89] Reay D., Smith P., van Amstel A. (2010). Methane and Climate Change.

